# Supplementary material for: First-principles phonon physics using the Pheasy code
Source: NPJ Comput Mater. 2026 Jun 24;12(1):343. doi: 10.1038/s41524-026-02163-1 (PMC13672059; doi:10.1038/s41524-026-02163-1)
Supplement: Supplementary file 1 [file 41524_2026_2163_MOESM1_ESM.pdf]

Supplementary information: First-principles phonon physics using the `Pheasy` code

Changpeng Lin,[1,*] Jian Han,[2] Ben Xu,[3,†] and Nicola Marzari[1,4]

[1] *Theory and Simulation of Materials (THEOS), and National Centre for Computational Design and Discovery of Novel Materials (MARVEL), École Polytechnique Fédérale de Lausanne, 1015 Lausanne, Switzerland*

[2] *State Key Laboratory of New Ceramics and Fine Processing, School of Materials Science and Engineering, Tsinghua University, 100084 Beijing, People's Republic of China*

[3] *Graduate School of China Academy of Engineering Physics, 100088 Beijing, People's Republic of China*

[4] *Laboratory for Materials Simulations, Paul Scherrer Institut, 5232 Villigen PSI, Switzerland*

(Dated: May 7, 2026)

Contents

Supplementary Note 1: General formulation of lattice dynamics 2

Supplementary Note 2: Harmonic and anharmonic phonon properties of silicon 4

Supplementary Note 3: Harmonic phonon properties of sodium chloride 6

Supplementary Note 4: Anharmonic phonon dispersion of Tl_3VSe_4 7

Supplementary Note 5: Additional figures and tables 8

Supplementary References 11

* cl834@cam.ac.uk; Present address: Department of Materials Science and Metallurgy, University of Cambridge, 27 Charles Babbage Road, Cambridge CB3 0FS, United Kingdom

† bxu@gscaep.ac.cn

Supplementary Note 1: General formulation of lattice dynamics

In this section, we review and provide the fundamental equations underlying the lattice dynamics of crystals within the harmonic approximation. Then, the expressions used in `Pheasy` for computing major harmonic and anharmonic properties are also summarized, including thermodynamic functions, thermal mean square displacement, mode participation ratio and Grüneisen parameter.

The equations of motion for atomic vibrations in crystalline solids can be readily derived from Newton's second law, and its secular equation in reciprocal space reads[1,2]:

$$\omega_\nu^2(\mathbf{q})e_{\nu,\kappa\alpha}(\mathbf{q}) = D_{\kappa\alpha,\kappa'\beta}(\mathbf{q})e_{\nu,\kappa'\beta}(\mathbf{q}), \tag{1}$$

where $D_{\kappa\alpha,\kappa'\beta}(\mathbf{q})$ is the dynamical matrix at a given wavevector $\mathbf{q}$, κ is the atom index within a unit cell, and α is the Cartesian component. If there are N_a atoms in the unit cell, by diagonalizing $D_{\kappa\alpha,\kappa'\beta}(\mathbf{q})$, one can obtain $3N_\mathrm{a}$ phonon modes labeled by the momentum $\mathbf{q}$ and band index ν, with the frequency $\omega_\nu(\mathbf{q})$ and the corresponding eigenvector $e_{\nu,\kappa\alpha}(\mathbf{q})$. Thus, $D_{\kappa\alpha,\kappa'\beta}(\mathbf{q})$ is the central quantity in a lattice-dynamical problem, which can be constructed via a Fourier transform of real-space interatomic force constants (IFCs) $\Phi_{\kappa\alpha,\kappa'\beta}(\mathbf{R})$ as

$$D_{\kappa\alpha,\kappa'\beta}(\mathbf{q}) = \sum_{\mathbf{R}} \frac{\Phi_{\kappa\alpha,\kappa'\beta}(\mathbf{R})}{\sqrt{m_\kappa m_{\kappa'}}} \mathrm{e}^{i\mathbf{q}\cdot\boldsymbol{\tau}_{\kappa\kappa'}(\mathbf{R})}, \tag{2}$$

where m_κ is the mass of the κth atom in the unit cell, $\boldsymbol{\tau}_{\kappa\kappa'}(\mathbf{R}) \equiv \boldsymbol{\tau}_{\kappa'}(\mathbf{R}) - \boldsymbol{\tau}_\kappa(\mathbf{0})$ is the relative distance between the κ'th atom of the unit cell $\mathbf{R}$ and the κth atom of the unit cell $\mathbf{0}$, $\boldsymbol{\tau}_{\kappa'}(\mathbf{R})$ and $\boldsymbol{\tau}_\kappa(\mathbf{0})$ are their respective positions, and $\mathbf{R}$ is the position vector of a unit cell. Thanks to the translation invariance of crystals, we have set the reference unit cell at $\mathbf{R} = \mathbf{0}$, and it is sufficient to consider the second-order IFCs $\Phi_{\kappa\alpha,\kappa'\beta}(\mathbf{R}) \equiv \Phi_{\kappa\alpha,\kappa'\beta}(\mathbf{0},\mathbf{R})$ between the atoms κ' in all unit cells $\{\mathbf{R}\}$ and the atoms κ in the reference unit cell. It is noteworthy that the definition of $D_{\kappa\alpha,\kappa'\beta}(\mathbf{q})$ depends on the adopted phase convention, and another common choice is

$$D_{\kappa\alpha,\kappa'\beta}(\mathbf{q}) = \sum_{\mathbf{R}} \frac{\Phi_{\kappa\alpha,\kappa'\beta}(\mathbf{R})}{\sqrt{m_\kappa m_{\kappa'}}} \mathrm{e}^{i\mathbf{q}\cdot\mathbf{R}}, \tag{3}$$

which only involves the unit cell position $\mathbf{R}$. The above two definitions are linked by a similarity transformation, and we adopt the latter case in the `Pheasy` code. It is important to keep a consistent phase convention, especially when the long-range contributions to dynamical matrix are present. `Pheasy` implements both the Ewald summation technique[3] and the mixed-space approach[4] to handle long-range electrostatic interactions in the long-wavelength limit. In the Ewald summation, we have considered long-range multipolar interactions up to the second order in phonon momentum $\mathbf{q}$, and see Ref.[5] for details; these include the zeroth-order dipole-dipole terms, the first-order dipole-quadrupole terms, and the second-order dipole-octupole, quadrupole-quadrupole and dielectric-dispersion-mediated dipole-dipole terms. By contrast, only the lowest-order dipole-dipole interactions are available in the mixed-space approach.

With phonon frequencies $\omega_\nu(\mathbf{q})$ and eigenvectors $\mathbf{e}_{\nu,\kappa}(\mathbf{q})$ at hand, we can calculate a plethora of harmonic phonon properties. The phonon density of states (DOS) $g(\omega)$ are defined as

$$g(\omega) = \frac{1}{N_\mathbf{q}} \sum_{\mathbf{q}\nu} \delta[\omega - \omega_\nu(\mathbf{q})], \tag{4}$$

where $\delta(\omega)$ is the Dirac delta function at the frequency ω, and $N_\mathbf{q}$ is the number of $\mathbf{q}$-points sampled in the reciprocal space. The atom-projected density of states (PDOS) $g_\kappa(\omega, \hat{\mathbf{n}})$ are further given by

$$g_\kappa(\omega, \hat{\mathbf{n}}) = \frac{1}{N_\mathbf{q}} \sum_{\mathbf{q}\nu} |\hat{\mathbf{n}} \cdot \mathbf{e}_{\nu,\kappa}(\mathbf{q})|^2 \delta[\omega - \omega_\nu(\mathbf{q})], \tag{5}$$

along the direction specified by the unit projection direction vector $\hat{\mathbf{n}}$. For practical calculations, we approximate the Dirac delta function with a Gaussian function as $\delta(\omega) \approx \frac{1}{\sqrt{2\pi}\sigma}\exp(-\omega^2/\sigma^2)$, and σ is a smearing parameter in frequency unit. Besides, the precise calculation of $\delta(\omega)$ can be achieved using the tetrahedron integration method that does not rely on any adjustable parameter, and we employ the improved tetrahedron method proposed by Kawamura et al.[6].

The displacement-displacement correlation function defined in Eq. (15) of the main text can be recast into[7]

$$\langle u_{\kappa\alpha}(\mathbf{R})u_{\kappa'\beta}(\mathbf{R}')\rangle = \frac{\hbar}{2N_\mathbf{q}} \sum_{\mathbf{q}\nu} \frac{2n_\nu(\mathbf{q},T)+1}{\omega_\nu(\mathbf{q})} \frac{e_{\nu,\kappa\alpha}(\mathbf{q})e^*_{\nu,\kappa'\beta}(\mathbf{q})}{\sqrt{m_\kappa m_{\kappa'}}} \mathrm{e}^{i\mathbf{q}\cdot(\mathbf{R}-\mathbf{R}')}, \tag{6}$$

where $\hbar$ is the reduced Planck constant, $n_\nu(\mathbf{q},T)=\frac{1}{\exp[\hbar\omega_\nu(\mathbf{q})/(k_\mathrm{B}T)]-1}$ is the Bose–Einstein distribution function at the given temperature T, and k_B is the Boltzmann constant. Then, the thermal mean square displacements (MSDs) for atoms in the reference unit cell $\mathbf{R}=\mathbf{0}$ can be readily obtained as

$$\left\langle \left| u_{\kappa\alpha} \right|^2 \right\rangle = \frac{\hbar}{2N_\mathbf{q} m_\kappa}\sum_{\mathbf{q}\nu}\frac{2n_\nu(\mathbf{q},T)+1}{\omega_\nu(\mathbf{q})}|e_{\nu,\kappa\alpha}(\mathbf{q})|^2, \tag{7}$$

which are just the diagonal elements of Eq. (6). Thermal MSDs can be further projected onto an arbitrary direction via the following expression:

$$\left\langle \left| u_{\kappa\alpha}(\hat{\mathbf{n}}) \right|^2 \right\rangle = \frac{\hbar}{2N_\mathbf{q} m_\kappa}\sum_{\mathbf{q}\nu}\frac{2n_\nu(\mathbf{q},T)+1}{\omega_\nu(\mathbf{q})}|\hat{\mathbf{n}}\cdot\mathbf{e}_{\nu,\kappa}(\mathbf{q})|^2. \tag{8}$$

The mode participation ratio $P_\nu(\mathbf{q})$ is an useful quantity to measure the localization of phonon modes in the system, originally defined by Bell and Dean[8]:

$$P_\nu(\mathbf{q}) = \frac{1}{N_\mathrm{a}\sum_\kappa|\mathbf{e}_{\nu,\kappa}(\mathbf{q})|^4}. \tag{9}$$

Specifically, $P_\nu(\mathbf{q})$ ranges from $1/N_\mathrm{a}$ to 1, representing the percentage of atoms participating in a given phonon mode. In other words, $P_\nu(\mathbf{q})$ is of $\mathcal{O}(1)$ when all atoms in the unit cell participate in such vibrational mode, and of $\mathcal{O}(1/N_\mathrm{a})$ when only one atom is involved. Additionally, in the literature, there exists another mass-weighted definition as[9]

$$P_\nu(\mathbf{q}) = \left(\sum_\kappa\frac{|\mathbf{e}_{\nu,\kappa}(\mathbf{q})|^2}{m_\kappa}\right)\bigg/\left(N_\mathrm{a}\sum_\kappa\frac{|\mathbf{e}_{\nu,\kappa}(\mathbf{q})|^4}{m_\kappa^2}\right). \tag{10}$$

If removing m_κ, it is easy to show that the numerator of Eq. (10) becomes unity, given the orthonormal conditions of eigenvectors: $\sum_\kappa \mathbf{e}_{\nu,\kappa}(\mathbf{q})\cdot\mathbf{e}^*_{\nu',\kappa}(\mathbf{q})=\delta_{\nu,\nu'}$, and Eq. (10) is hence reduced to Eq. (9). As shown in Supplementary Figure **3**(d) for NaCl, these two definitions generally yield different results for any compound with multiple elements, and they are only equivalent to each other for monoelemental solids [see Supplementary Figure **2** (d) for silicon].

The vibrational thermodynamic properties, including harmonic internal energy U, constant-volume heat capacity C_V, Helmholtz free energy F and entropy S, are calculated via the following expressions[2]:

$$U = \frac{1}{N_\mathbf{q}}\sum_{\mathbf{q}\nu}\hbar\omega_\nu(\mathbf{q})\left[\frac{1}{2}+\frac{1}{\mathrm{e}^{\hbar\omega_\nu(\mathbf{q})/(k_\mathrm{B}T)}-1}\right], \tag{11}$$

$$C_V \equiv \left(\frac{\partial U}{\partial T}\right)_V = \frac{k_\mathrm{B}}{N_\mathbf{q}}\sum_{\mathbf{q}\nu}\left[\frac{\hbar\omega_\nu(\mathbf{q})}{2k_\mathrm{B}T}\right]^2\mathrm{cosech}^2\left[\frac{\hbar\omega_\nu(\mathbf{q})}{2k_\mathrm{B}T}\right], \tag{12}$$

$$F \equiv -k_\mathbf{B}T\ln Z = \frac{k_\mathbf{B}T}{N_\mathbf{q}}\sum_{\mathbf{q}\nu}\left[\ln\left(2\sinh\left[\frac{\hbar\omega_\nu(\mathbf{q})}{2k_\mathrm{B}T}\right]\right)\right], \tag{13}$$

$$S \equiv -\frac{\partial F}{\partial T} = \frac{k_\mathrm{B}}{N_\mathbf{q}}\sum_{\mathbf{q}\nu}\left\{\frac{\hbar\omega_\nu(\mathbf{q})}{2k_\mathrm{B}T}\frac{1}{\mathrm{e}^{\hbar\omega_\nu(\mathbf{q})/(k_\mathrm{B}T)}-1}-\ln\left[1-\mathrm{e}^{-\hbar\omega_\nu(\mathbf{q})/(k_\mathrm{B}T)}\right]\right\}, \tag{14}$$

where Z is the partition function.

The mode Grüneisen parameter is defined as[2]

$$\gamma_\nu(\mathbf{q}) \equiv -\frac{\partial\ln\omega_\nu(\mathbf{q})}{\partial\ln\Omega} = -\frac{\Omega}{\omega_\nu(\mathbf{q})}\frac{\partial\omega_\nu(\mathbf{q})}{\partial\Omega}, \tag{15}$$

with the unit cell volume Ω. Using perturbation theory, $\gamma_\nu(\mathbf{q})$ can be obtained easily via cubic IFCs as[10]:

$$\gamma_\nu(\mathbf{q}) = -\frac{1}{6\omega_\nu^2(\mathbf{q})}\sum_{\mathbf{R}\mathbf{R}'}\sum_{\kappa\kappa'\kappa''}\sum_{\alpha\beta\gamma}\frac{e_{\nu,\kappa\alpha}(\mathbf{q})e^*_{\nu,\kappa'\beta}(\mathbf{q})}{\sqrt{m_\kappa m_{\kappa'}}}\Phi_{\kappa\alpha,\kappa'\beta,\kappa''\gamma}(\mathbf{R},\mathbf{R}')\tau_{\kappa''\gamma}(\mathbf{R}')\mathrm{e}^{i\mathbf{q}\cdot\mathbf{R}}, \tag{16}$$

where $\Phi_{\kappa\alpha,\kappa'\beta,\kappa''\gamma}(\mathbf{R},\mathbf{R}')\equiv\Phi_{\kappa\alpha,\kappa'\beta,\kappa''\gamma}(\mathbf{0},\mathbf{R},\mathbf{R}')$ are the third-order IFCs. With the mode-dependent heat capacity $C_\nu(\mathbf{q})$, the thermodynamic Grüneisen parameter is further calculated as[2]

$$\gamma = \sum_{\mathbf{q}\nu}\frac{\gamma_\nu(\mathbf{q})C_\nu(\mathbf{q})}{C_V}. \tag{17}$$

Supplementary Note 2: Harmonic and anharmonic phonon properties of silicon

Here, we perform additional benchmarks on bulk silicon to validate the accuracy of `Pheasy`'s implementation of basic harmonic and anharmonic properties summarized in the Supplementary Note 1. Computational details can be found in the "Method" section of the main text. We calculate harmonic IFCs of silicon using the small-displacement method, where 5 displaced supercells are generated via randomly moving all atoms with a magnitude of 0.01 Å, as compared to the results from `Phonopy`[11–13]. Similarly, we create 20 randomly displaced configurations with a displacement magnitude of 0.03 Å to obtain the third-order IFCs of silicon, which are further compared with those from `thirdorder.py`[14] to calculate lattice thermal conductivity (LTC) using `ShengBTE`[14].

In Supplementary Figure 1, we compare our calculated mode Grüneisen parameters, three-phonon scattering rates and LTCs of silicon with the results using cubic IFCs from `thirdorder.py`. An overall good agreement is achieved with just 10 displacements in `Pheasy`, while conventional finite-displacement approach in `thirdorder.py` requires 124 displaced structures, i.e. a speed-up of ∼12 folds. Furthermore, we plot the phonon spectra, density of states and related harmonic and anharmonic properties of silicon in Supplementary Figure 2. Our results using `Pheasy` are in excellent agreement with those calculated from `Phonopy`.

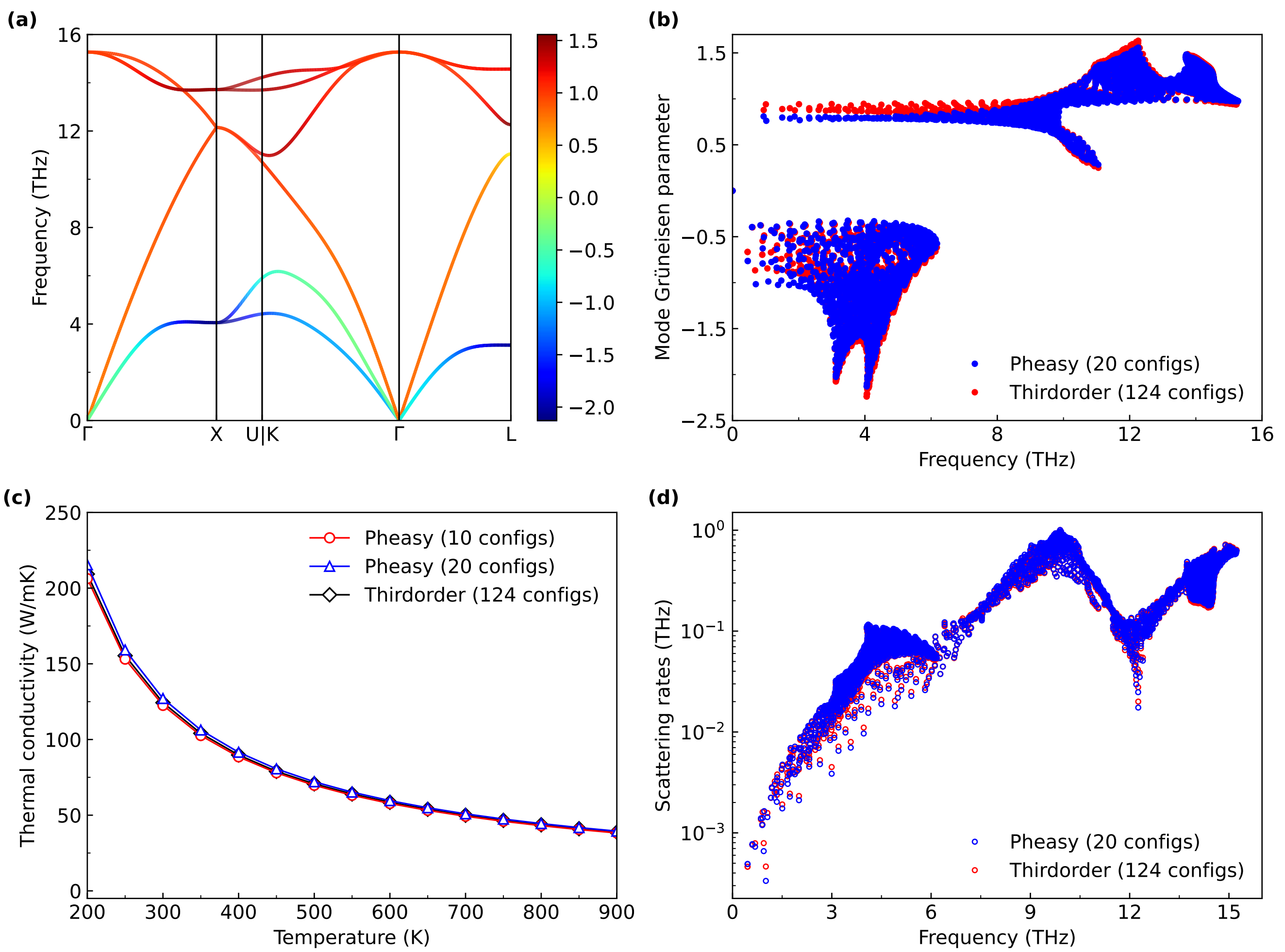

Supplementary Figure 1. Grüneisen parameters and thermal transport properties of silicon: (a) phonon dispersions with colors denoting the values of mode Grüneisen parameters; (b) mode Grüneisen parameters as a function of phonon frequencies; (c) temperature-dependent LTCs from 200 to 900 K; (d) three-phonon scattering rates as a function of phonon frequencies. Comparisons are drawn between the results using the third-order IFCs from `Pheasy` and `thirdorder.py`[14].

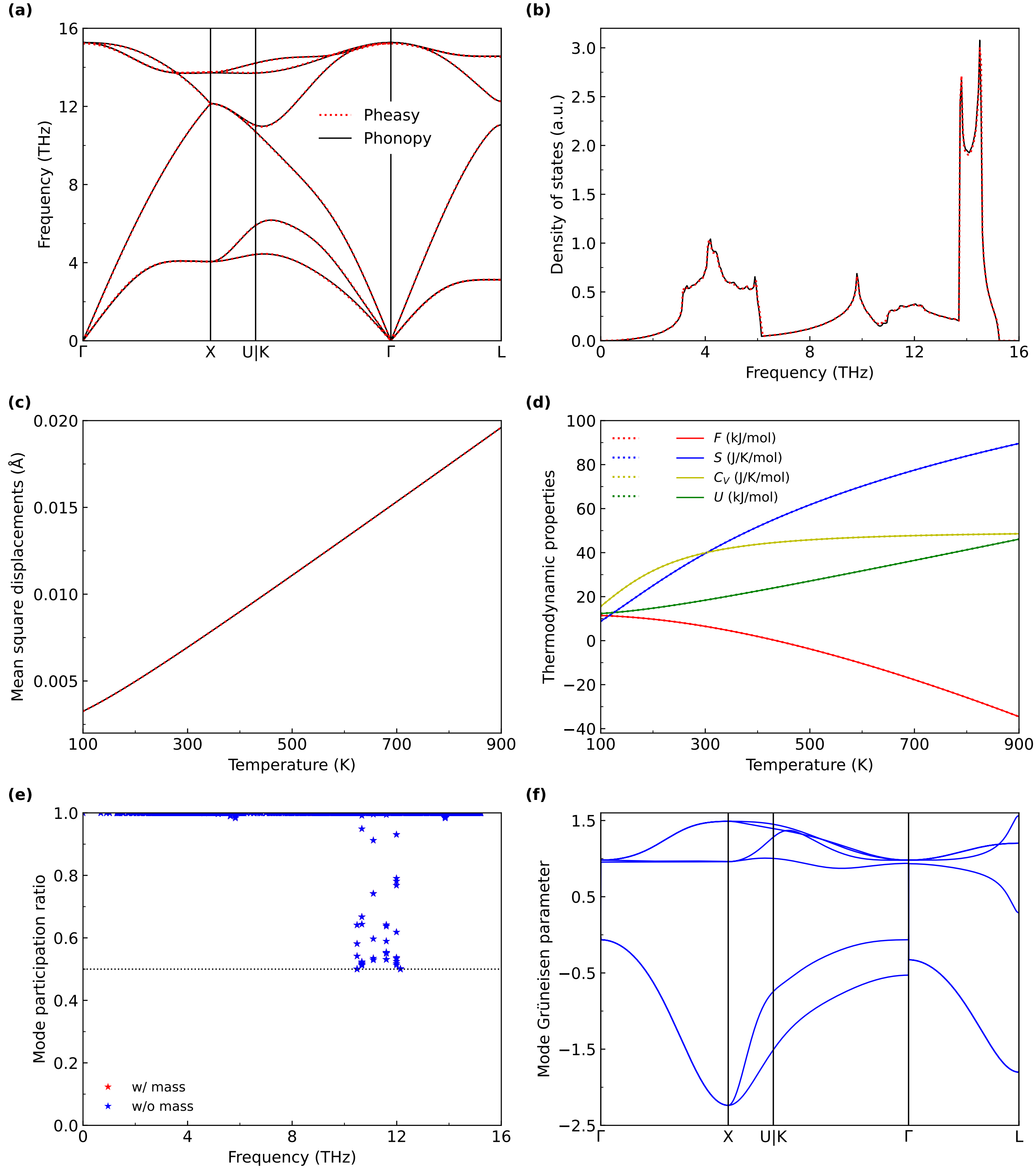

Supplementary Figure 2. Harmonic and anharmonic phonon properties of silicon: (a) phonon dispersions; (b) density of states; (c) thermal mean square displacements as a function of temperature; (d) vibrational thermodynamic properties as a function of temperature, including free energy (F), entropy (S), constant-volume heat capacity (C_V) and internal energy (U); (e) mode participation ratios as a function of phonon frequencies using two different definitions; (f) mode Grüneisen parameter along the high-symmetry paths. Solid and dotted lines are the results calculated using `Phonopy`[11–13] and `Pheasy`, respectively.

Supplementary Note 3: Harmonic phonon properties of sodium chloride

We choose bulk sodium chloride (NaCl) to validate the correctness of our implementation for long-range Coulomb interactions in the `Pheasy` code. DFT calculations are performed using the `Quantum ESPRESSO`[15,16], with the norm-conserving pseudopotentials from PseudoDojo[17]. We adopt the Perdew, Burke and Ernzerhof's (PBE) parametrization[18] of the generalized gradient approximation (GGA) for describing the exchange and correlation effects of electrons. The kinetic energy cutoff is set to 160 Ry, and a $12 \times 12 \times 12$ electronic grid is used to sampling the Brillouin zone. Using the same structure optimization criteria as specified in the "Methods" section of the main text, we obtain an optimized lattice constant of NaCl as 5.698 Å. To calculate the real-space IFCs, we use a $3 \times 3 \times 3$ supercell (54 atoms) for the real-space small displacement methods in `Pheasy` and `Phonopy`, which corresponds to a $3 \times 3 \times 3$ **q**-grid in reciprocal-space DFPT calculations. With `Pheasy`, we generate 5 randomly displaced structures with a displacement magnitude of 0.01 Å for all atoms, and we find that 2 configurations are enough to converge harmonic IFCs. Then, DFT calculations with a $2 \times 2 \times 2$ **k**-grid are performed for all supercells to get their interatomic forces, while DFPT calculations are executed with a $6 \times 6 \times 6$ **k**-grid to ensure the consistency of computational setup. The Born effective charge and dielectric tensors of NaCl are also obtained via DFPT, with a denser $12 \times 12 \times 12$ **k**-grid.

The calculated phonon dispersions, atom-projected density of states and related harmonic properties of NaCl are shown in Supplementary Figure **3**. Our results from `Pheasy` are in excellent agreement with those from `Quantum ESPRESSO` and `Phonopy`.

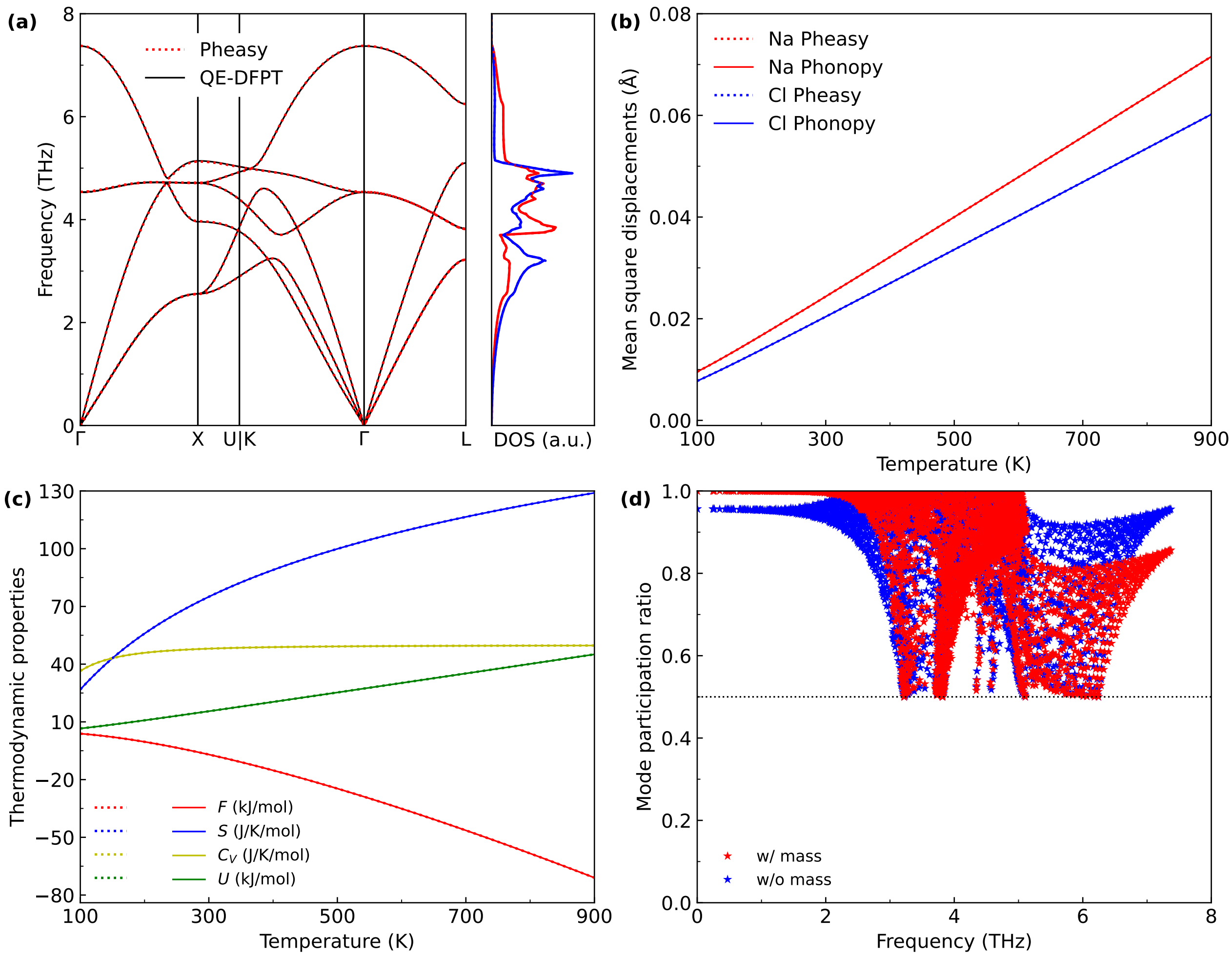

Supplementary Figure 3. Harmonic phonon properties of NaCl: (a) phonon dispersions and atom-projected density of states; (b) thermal mean square displacements as a function of temperature; (c) vibrational thermodynamic properties as a function of temperature, including free energy (F), entropy (S), constant-volume heat capacity (C_V) and internal energy (U); (d) mode participation ratios as a function of phonon frequencies using two different definitions. Solid and dotted lines are the results calculated using `Phonopy`[11–13] [or `Quantum ESPRESSO` (QE)[15,16]] and `Pheasy`, respectively.

Supplementary Note 4: Anharmonic phonon dispersion of Tl_3VSe_4

To identify the optimal scheme for IFC extraction, here we further use Tl_3VSe_4 as an example to show that the one-shot approach outperforms the cocktail method [see point (v) of our general guidelines in the main text for details]. All DFT and DFPT calculations are performed using the `Quantum ESPRESSO` package[15,16], with the exchange-correlation functionals and pseudopotentials as described in the "Methods" section of the main text. The plane-wave energy cutoffs are set to 90 and 900 Ry, respectively, for the expansion of wavefunction and charge density. While we use a $12 \times 12 \times 12$ electronic grid for single unit cell calculations, a $2 \times 2 \times 2$ grid is adopted for all supercell calculations. To extract the high-order IFCs of Tl_3VSe_4 up to sixth order, we construct a $2 \times 2 \times 2$ supercell with cutoffs of the seventh and third nearest neighbors for the third-order and the remaining higher-order IFCs, respectively. These truncations correspond to the two cutoff distances of 7.14 and 4.88 Å, which has been shown sufficient to converge the LTC of Tl_3VSe_4[19]. Moreover, for quartic IFCs, we neglect the insignificant four-body interactions and keep only the on-site and two-body terms in the fifth- and sixth-order IFCs. The resulting numbers of independent IFC parameters from second to sixth order are 123, 2126, 489, 63 and 90, respectively. Similar to the example of cubic $SrTiO_3$ in the main text, the training dataset is prepared using the finite-temperature displacements at 50 K as implemented in the SCHA method[20,21], where each ensemble has at least 64 displaced configurations.

We use the room-temperature phonon dispersion from the SCHA method as a reference to benchmark the accuracy of different flavors to extract high-order IFCs. As shown in Supplementary Figure 4, the SCP calculation with the fourth-order IFCs from the one-shot scheme yields a phonon spectrum in good overall agreement with the SCHA results, whereas the one from the cocktail flavor leads to overestimated frequencies of most phonon branches. The harmonic IFCs kept fixed during the cocktail fitting are derived from a DFPT calculation on a primitive unit cell, using phonon and electronic grids of $2 \times 2 \times 2$ and $4 \times 4 \times 4$, respectively. Such a DFPT calculation is consistent with the computational setup in real space employing a $2 \times 2 \times 2$ supercell and a $2 \times 2 \times 2$ $\mathbf{k}$-grid.

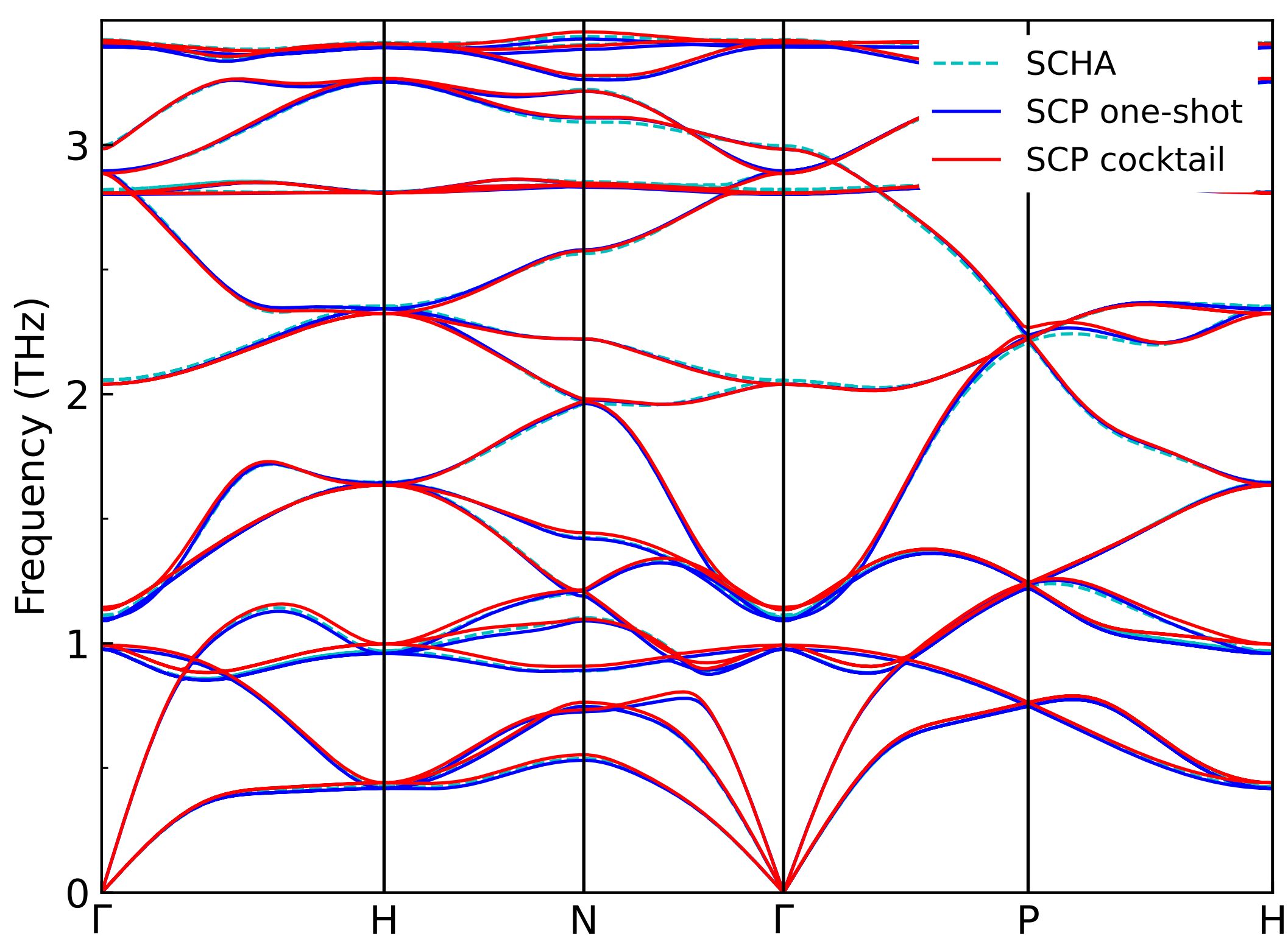

Supplementary Figure 4. Room-temperature phonon dispersions of Tl_3VSe_4 calculated using the SCHA and SCP approaches. The one-shot scheme extracts all-order IFCs simultaneously during fitting, whereas the cocktail method fits only the anharmonic IFCs, keeping the harmonic terms fixed to the DFPT results.

Supplementary Note 5: Additional figures and tables

Supplementary Table 1. Lattice thermal conductivity of bulk silicon and monolayer WS_2 at 300 K calculated with the increasing hierarchy of thermal transport theory, and their self-consistency using different ensembles. We start from the lowest harmonic approximation (HA) to obtain phonon spectra and include only three-phonon (3ph) scattering. The complexity of thermal transport theory increases by taking into account temperature-dependent phonon frequencies (via self-consistent phonons, i.e. SCP) and anharmonic interatomic force constants (IFCs), as well as adding four-phonon (4ph) scattering. All thermal conductivities of silicon and 2D WS_2 are calculated based on a $30 \times 30 \times 30$ and a $90 \times 90 \times 1$ **q**-mesh, respectively. The iterative solution of phonon Boltzmann transport equation is achieved only for 3ph scattering, while 4ph scattering is obtained within the relaxation time approximation (RTA). A sampling method[22] using 10^5 processes is further used to accelerate the estimation of 4ph scattering within RTA, and the thickness of 2D WS_2 is set to 6.16 Å[23]. The force-displacement dataset was generated using the quantum canonical sampling of normal modes at 300 K, and there are 64 configurations in each ensemble.

Materials	Ensemble No.	HA+3ph	HA+3,4ph	SCP+3ph	SCP+3,4ph	SCP+3ph+IFCs@T	SCP+3,4ph+IFCs@T
Silicon	1	124.85	115.13	122.49	112.48	125.90	115.32
	2	125.40	115.54	122.33	112.18	124.57	114.18
WS_2	1	243.69	206.43	241.90	205.87	238.39	201.05
	2	241.61	202.48	239.68	201.71	239.10	202.70

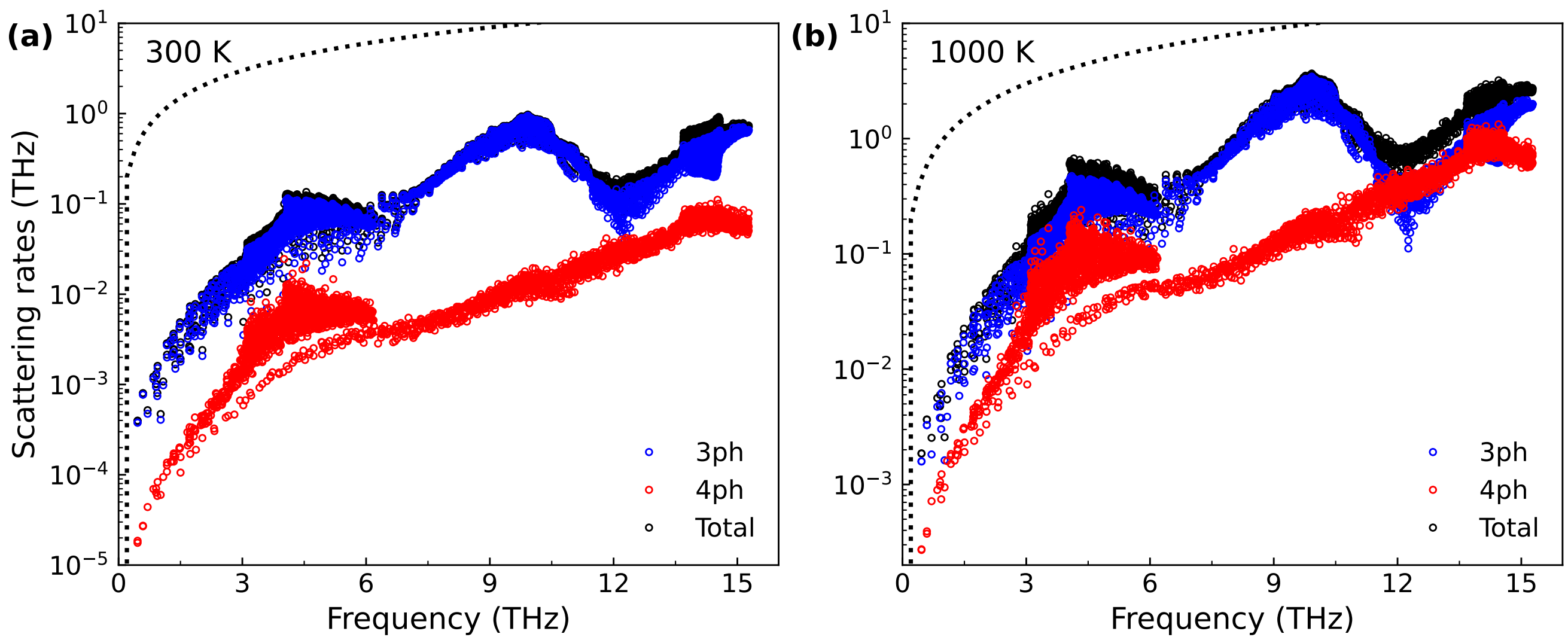

Supplementary Figure 5. Three- and four-phonon scattering rates as a function of phonon frequencies in silicon at (a) 300 and (b) 1000 K. The dotted line indicates where phonon frequency equals scattering rate, i.e. the breakdown of quasiparticle approximation.

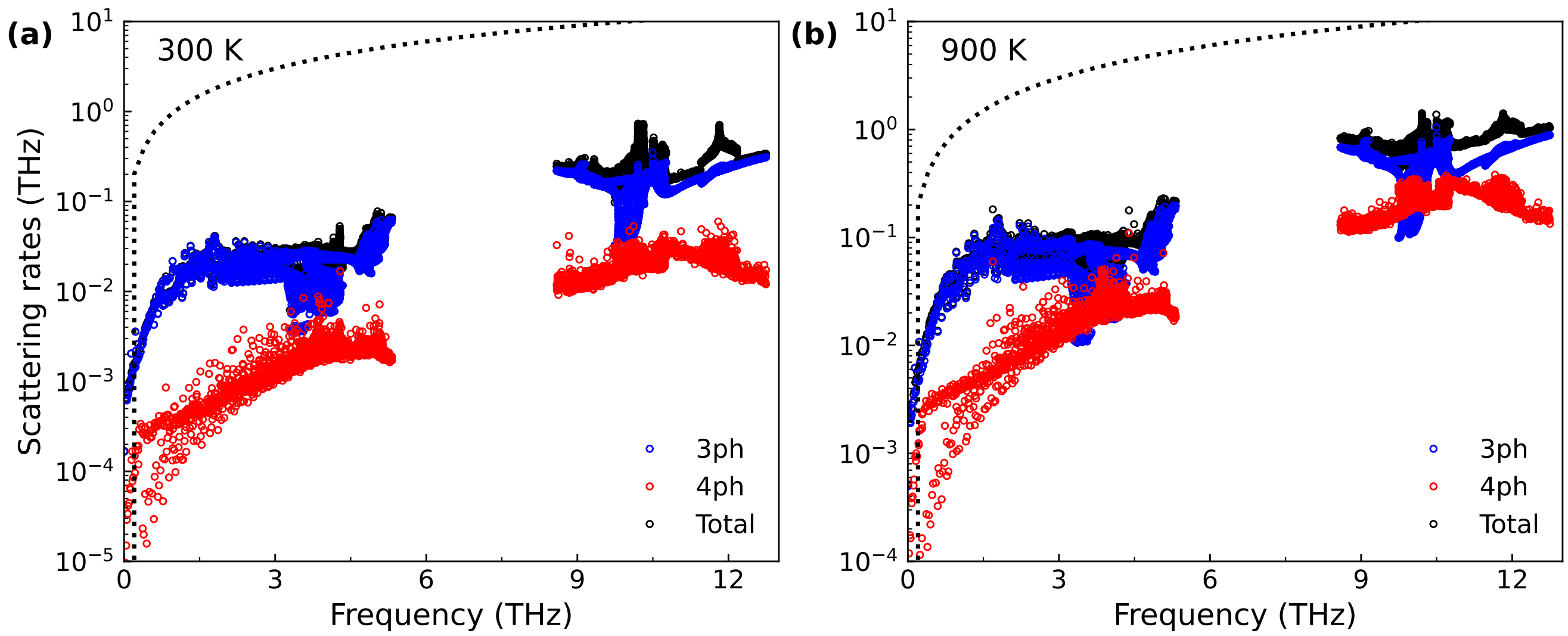

Supplementary Figure 6. Three- and four-phonon scattering rates as a function of phonon frequencies in WS_2 monolayer at (a) 300 and (b) 900 K. The dotted line indicates where phonon frequency equals scattering rate, i.e. the breakdown of quasiparticle approximation.

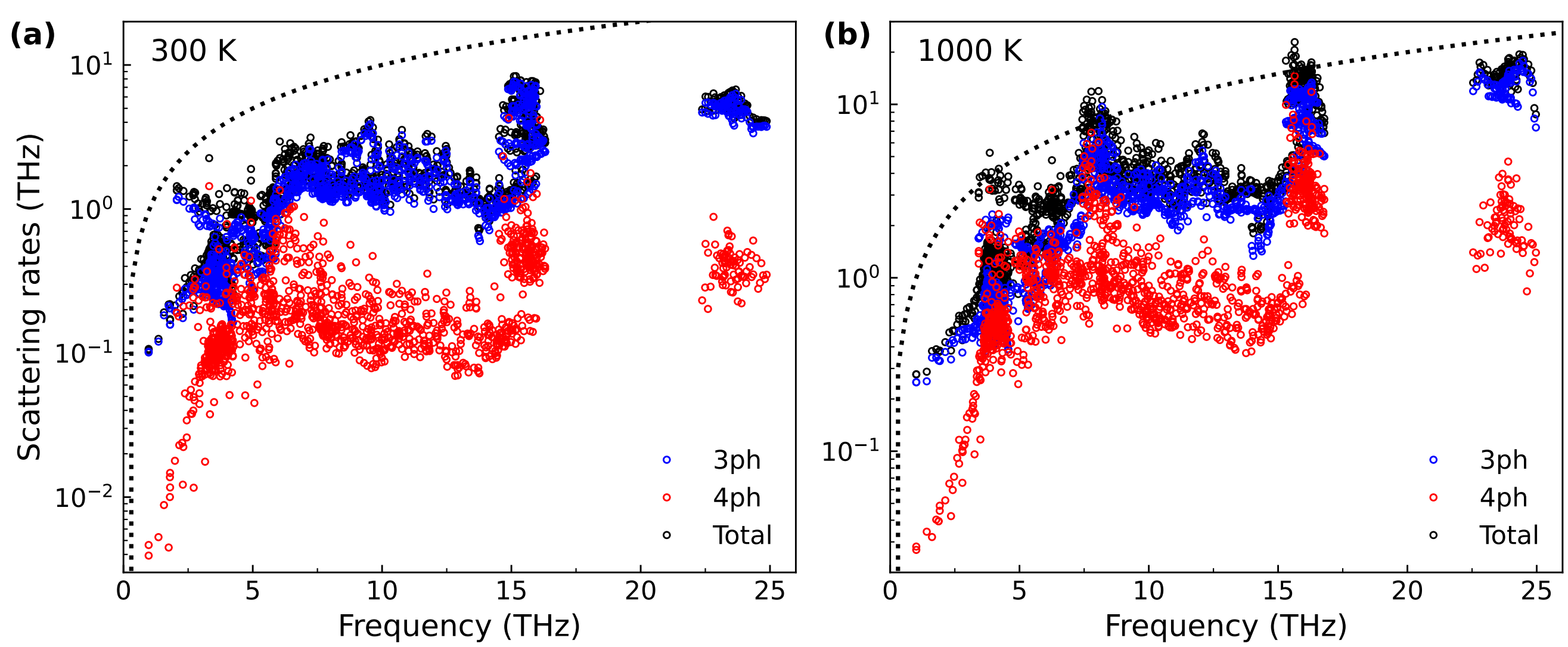

Supplementary Figure 7. Three- and four-phonon scattering rates as a function of phonon frequencies in cubic $SrTiO_3$ at (a) 300 and (b) 1000 K. The dotted line indicates where phonon frequency equals scattering rate, i.e. the breakdown of quasiparticle approximation.

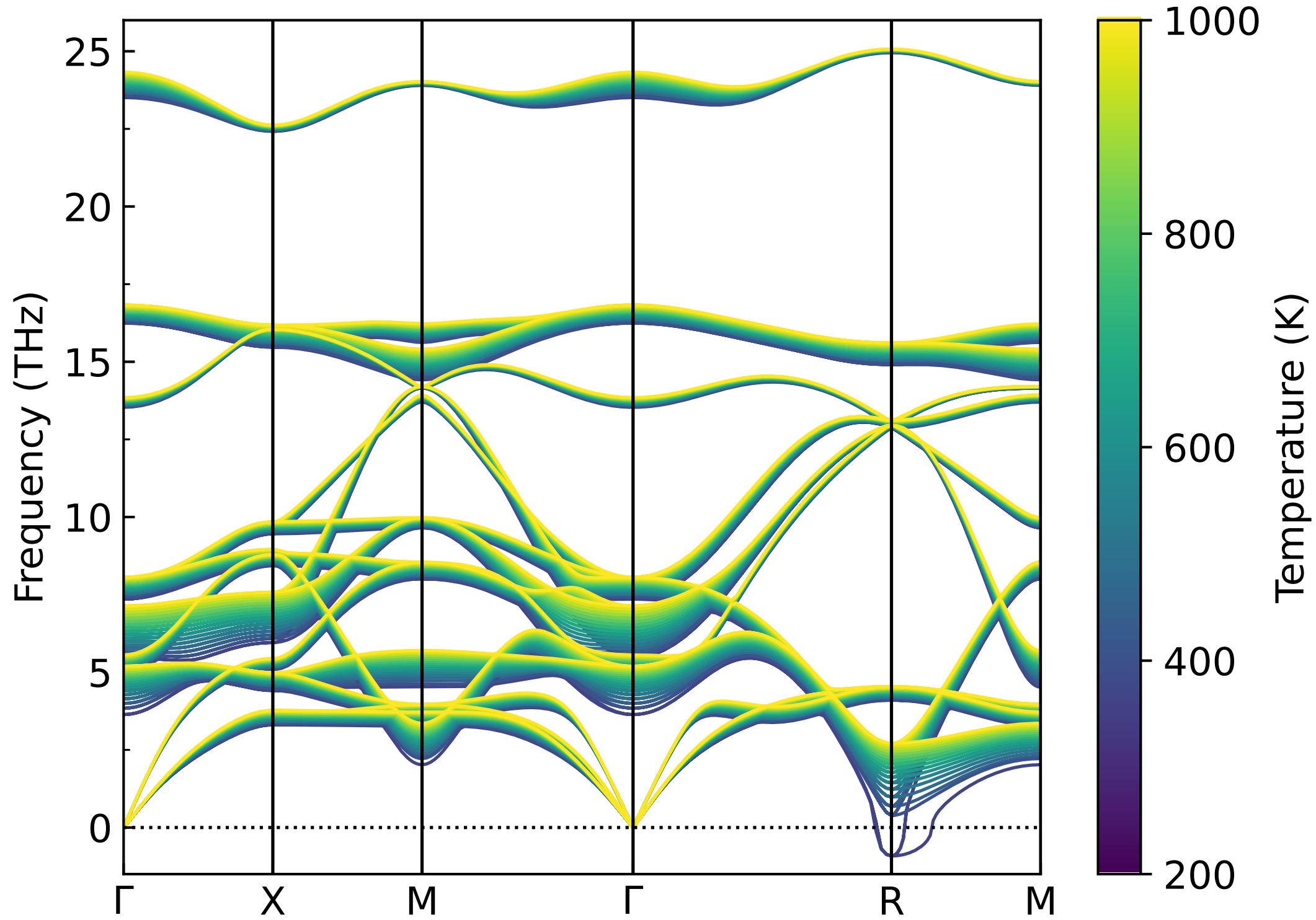

Supplementary Figure 8. Temperature-dependent phonon dispersions of cubic $SrTiO_3$ from 200 to 1000 K, calculated using the SCP approach with the loop diagram of phonon self-energy. The SCP equation is solved on a $12 \times 12 \times 12$ **q**-grid, which suggests a phase-transition temperature between 200 and 250K, in agreement with the SCP calculations by Tadano et al.[24].

Supplementary References

1. Born, M. & Huang, K. *Dynamical Theory of Crystal Lattices* (Oxford University Press, 1954). URL http://dx.doi.org/10.1093/oso/9780192670083.001.0001.
2. Dove, M. T. *Introduction to Lattice Dynamics* (Cambridge University Press, Cambridge, 1993).
3. Gonze, X. & Lee, C. Dynamical Matrices, Born Effective Charges, Dielectric Permittivity Tensors, and Interatomic Force Constants from Density-Functional Perturbation Theory. *Phys. Rev. B* **55**, 10355–10368 (1997).
4. Wang, Y. *et al.* A Mixed-Space Approach to First-Principles Calculations of Phonon Frequencies for Polar Materials. *J. Phys.: Condens. Matter* **22**, 202201 (2010). URL http://dx.doi.org/10.1088/0953-8984/22/20/202201.
5. Lin, C., Poncé, S., Macheda, F., Mauri, F. & Marzari, N. Elastic Constants and Bending Rigidities from Long-Wavelength Perturbation Expansions. *PRX Energy* **5**, 013012 (2026). URL http://dx.doi.org/10.1103/hc53-g1p3.
6. Kawamura, M., Gohda, Y. & Tsuneyuki, S. Improved Tetrahedron Method for the Brillouin-Zone Integration Applicable to Response Functions. *Phys. Rev. B* **89**, 094515 (2014). URL http://dx.doi.org/10.1103/PhysRevB.89.094515.
7. Wallace, D. C. *Thermodynamics of Crystals* (John Wiley & Sons, 1972). URL https://books.google.ch/books?id=HHqLPwAACAAJ.
8. Bell, R. J. & Dean, P. Atomic Vibrations in Vitreous Silica. *Discuss. Faraday Soc.* **50**, 55–61 (1970). URL http://dx.doi.org/10.1039/DF9705000055.
9. Hafner, J. & Krajci, M. Propagating and confined vibrational excitations in quasicrystals. *J. Phys.: Condens. Matter* **5**, 2489–2510 (1993). URL http://dx.doi.org/10.1088/0953-8984/5/16/008.
10. Fabian, J. & Allen, P. B. Thermal Expansion and Grüneisen Parameters of Amorphous Silicon: A Realistic Model Calculation. *Phys. Rev. Lett.* **79**, 1885–1888 (1997). URL http://dx.doi.org/10.1103/PhysRevLett.79.1885.
11. Togo, A. & Tanaka, I. First Principles Phonon Calculations in Materials Science. *Scr. Mater.* **108**, 1–5 (2015). URL http://dx.doi.org/10.1016/j.scriptamat.2015.07.021.
12. Togo, A. First-Principles Phonon Calculations with Phonopy and Phono3py. *J. Phys. Soc. Japan* **92**, 012001 (2023). URL http://dx.doi.org/10.7566/JPSJ.92.012001.
13. Togo, A., Chaput, L., Tadano, T. & Tanaka, I. Implementation Strategies in Phonopy and Phono3py. *J. Phys.: Condens. Matter* **35**, 353001 (2023). URL http://dx.doi.org/10.1088/1361-648X/acd831.
14. Li, W., Carrete, J., A. Katcho, N. & Mingo, N. Shengbte: A Solver of the Boltzmann Transport Equation for Phonons. *Comput. Phys. Commun.* **185**, 1747–1758 (2014). URL http://dx.doi.org/10.1016/j.cpc.2014.02.015.
15. Giannozzi, P. *et al.* Quantum ESPRESSO: A Modular and Open-Source Software Project for Quantum Simulations of Materials. *J. Phys.: Condens. Matter* **21**, 395502 (2009). URL http://dx.doi.org/10.1088/0953-8984/21/39/395502.
16. Giannozzi, P. *et al.* Advanced Capabilities for Materials Modelling with Quantum Espresso. *J. Phys.: Condens. Matter* **29**, 465901 (2017). URL http://dx.doi.org/10.1088/1361-648x/aa8f79.
17. van Setten, M. *et al.* The PseudoDojo: Training and Grading a 85 Element Optimized Norm-Conserving Pseudopotential Table. *Comput. Phys. Commun.* **226**, 39–54 (2018). URL http://dx.doi.org/10.1016/j.cpc.2018.01.012.
18. Perdew, J. P., Burke, K. & Ernzerhof, M. Generalized Gradient Approximation Made Simple. *Phys. Rev. Lett.* **77**, 3865–3868 (1996). URL http://dx.doi.org/10.1103/PhysRevLett.77.3865.
19. Li, Z., Xia, Y. & Wolverton, C. First-Principles Calculations of Lattice Thermal Conductivity in Tl_3VSe_4: Uncertainties from Different Approaches of Force Constants. *Phys. Rev. B* **108**, 184307 (2023). URL http://dx.doi.org/10.1103/PhysRevB.108.184307.
20. Monacelli, L. *et al.* The stochastic self-consistent harmonic approximation: Calculating vibrational properties of materials with full quantum and anharmonic effects. *J. Phys.: Condens. Matter* **33**, 363001 (2021).
21. Lin, C. *Thermodynamics and Exotic Transport Phenomena in Emerging Materials*. Ph.D. thesis, EPFL (2025). URL https://infoscience.epfl.ch/handle/20.500.14299/256622.
22. Guo, Z., Han, Z., Feng, D., Lin, G. & Ruan, X. Sampling-Accelerated Prediction of Phonon Scattering Rates for Converged Thermal Conductivity and Radiative Properties. *npj Comput. Mater.* **10**, 31 (2024). URL http://dx.doi.org/10.1038/s41524-024-01215-8.
23. Schutte, W., De Boer, J. & Jellinek, F. Crystal Structures of Tungsten Disulfide and Diselenide. *J. Solid State Chem.* **70**, 207–209 (1987). URL http://dx.doi.org/10.1016/0022-4596(87)90057-0.
24. Tadano, T. & Tsuneyuki, S. Self-Consistent Phonon Calculations of Lattice Dynamical Properties in Cubic $SrTiO_3$ with First-Principles Anharmonic Force Constants. *Phys. Rev. B* **92**, 054301 (2015). URL http://dx.doi.org/10.1103/PhysRevB.92.054301.
